# Exercise-induced cardiac remodeling in farm dogs: A comparative echocardiographic and electrocardiographic study

**DOI:** 10.5455/javar.2026.m1055

**Published:** 2026-06-22

**Authors:** Nantawan Yatbantoong, Nattaphol Muangthong, Soontaree Petchdee

**Affiliations:** 1 Department of Large Animal and Wildlife Clinical Science, Kasetsart University, Nakhon Pathom, Thailand; 2 Kasetsart University Veterinary Teaching Hospital Kamphaeng Saen Campus, Faculty of Veterinary Medicine, Kasetsart University, Kamphaeng Saen Campus, Nakhon Pathom 73140, Thailand

**Keywords:** Dogs, echocardiography, eccentric remodeling, exercise, murmur

## Abstract

**Objectives:** To compare cardiac structure and function of healthy indoor dogs, healthy active farm dogs, and indoor dogs with structural heart disease, and to investigate whether cardiovascular differences are lifestyle-related.

**Materials and Methods:** Thirty-nine client-owned dogs (1–15 years old) were enrolled between January and March 2026. Dogs were grouped into healthy indoor dogs (*n* = 10), active farm dogs (*n* = 14), and indoor dogs with structural heart disease (*n* = 15). Physical exam, electrocardiography, and transthoracic echocardiography were performed in all dogs. Continuous variables were tested for statistical significance, with *p* < 0.05 indicating significance.

**Results:** Active farm dogs had significantly reduced resting heart rates in comparison to healthy indoor and diseased indoor dogs (*p* < 0.05). Echocardiography showed that farm dogs had larger normalized left ventricular inner measurements and smaller relative wall thickness than that in healthy indoor dogs (*p* < 0.05), indicating eccentric remodeling. However, the farm dogs were also significantly younger and heavier (*p* < 0.05). Dogs with heart disease had significantly greater left atrial size and differed in functional indices from those in the healthy group (*p* < 0.05). Cardiac murmurs occurred in all groups.

**Conclusions:** Cardiac morphology and functional features differed among dogs with different lifestyles and disease severity. Active farm dogs showed signs of physiological eccentric remodeling, although this might have been moderated by age, body size, and breed. Echocardiography remains essential for differentiating structural heart disease from physiological variation. Further study is required on the different effects of exercise on cardiac remodeling in dogs.

## 1. Introduction

Long-term physical activity can induce structural and functional changes in the cardiovascular system. In dogs, sustained exercise and regular physical work may lead to adaptive cardiac remodeling that supports increased circulatory demand during activity [1]. These physiological adaptations resemble the phenomenon often described as the athlete’s heart, which has been reported in several animal species exposed to repeated endurance exercise [2].

One of the principal adaptations to prolonged exercise is the modification of cardiac size and geometry. In active dogs, increased preload during exercise can lead to enlargement of the left ventricular chamber and an increase in ventricular mass. These changes are generally considered a response to chronic volume loading associated with endurance-type activity [3]. In contrast, activities involving short bursts of high intensity may promote increased ventricular wall thickness rather than chamber enlargement. Such adaptations help the heart maintain an adequate stroke volume during periods of increased metabolic demand.

Exercise training also affects cardiovascular function. Dogs that undergo regular physical activity may exhibit lower resting heart rates compared with sedentary animals. This reduction in heart rate is thought to result from enhanced vagal tone and improved myocardial efficiency [4]. At the same time, stroke volume typically increases due to improved ventricular filling and myocardial compliance. As a result, cardiac output during rest often remains relatively stable, whereas the ability to augment cardiac output during exercise is enhanced.

Repeated exercise is also associated with improvements in peripheral circulation. Increased capillary density in skeletal muscle and improved vascular recruitment facilitate greater blood flow to working tissues. In addition, overall blood volume may increase, contributing to improved oxygen delivery during activity. These cardiovascular adjustments allow active dogs to sustain higher levels of physical performance while maintaining stable systemic hemodynamics.

The structural and functional adaptations associated with regular exercise are generally considered physiological and reversible. However, distinguishing exercise-related cardiac remodeling from pathological cardiac disease remains clinically important. Certain cardiac disorders in dogs, such as dilated cardiomyopathy or myxomatous mitral valve disease, may also be associated with chamber enlargement or changes in ventricular function [5, 6, 7, 8]. Therefore, careful cardiovascular evaluation is required to differentiate normal adaptive remodeling from underlying diseases. Accurate interpretation of these findings is particularly relevant when examining active or working dogs that may present with cardiac murmurs or altered echocardiographic measurements despite being clinically healthy.

The objectives of this study were to compare cardiac structure and function among healthy indoor dogs with limited activity, healthy farm dogs with regular daily exercise, and indoor dogs with structural heart disease.

## 2. Materials and Methods

### 2.1. Ethical approval

The study was approved by Kasetsart University (ACKU69-VET-033), and written authorization from the owners permitting the use of their pets’ medical information for research was obtained at the time of routine health assessment.

### 2.2. Inclusion criteria

Client-owned dogs aged 1 to 15 years were included in this study. The study was conducted between January and March 2026, and all data were prospectively collected during this period. Animals with known systemic illness were excluded, and all enrolled dogs were free of clinical signs of cardiac disease based on owner history at the time of examination. Each dog underwent a single echocardiography classification based on established veterinary cardiology guidelines. They were presented during the study period for routine health assessment or cardiovascular screening. Only dogs that had a complete physical examination, electrocardiographic evaluation, and transthoracic echocardiography were included in the analysis. Dogs were divided into three groups based on lifestyle information and echocardiographic findings. Healthy indoor dogs (control group), active farm dogs engaged in daily physical activity (healthy farm dogs), and indoor dogs with echocardiographic evidence of structural heart disease (diseased indoor dogs). All included animals presented stable clinical status at examination and had adequate echocardiographic image quality to assess cardiac parameters.

### 2.3. Physical examination

Cardiac auscultation was performed by a single investigator (SP) who was unaware of any prior cardiac history reported by the owner. Each dog was examined while standing calmly with the owner-handler. Heart rate and the presence of cardiac murmurs were recorded. Murmurs were described according to timing (systolic or diastolic), intensity (graded 1–6, where grade 1 was the faintest audible murmur and grade 6 was audible with the stethoscope slightly lifted from the thoracic wall), and point of maximal intensity (left or right apex; left or right heart base). Auscultation findings were not disclosed to the echocardiographer until completion of the imaging study.

### 2.4. Electrocardiographic examination

A standard electrocardiogram (ECG) was recorded in all dogs using a multi-lead ECG system. Recordings were obtained with the dogs gently restrained in right lateral recumbency without sedation. Standard limb leads (I, II, III, aVR, aVL, and aVF) were recorded at a paper speed of 50 mm/sec and a calibration of 10 mm = 1 mV. Heart rhythm, heart rate, and conduction patterns were assessed from lead II. Arrhythmias, including supraventricular and ventricular premature complexes, were noted when present. Measurements included P wave duration and amplitude, PR interval, QRS duration, and R wave amplitude. The mean electrical axis was estimated using standard limb leads. All ECG tracings were evaluated by a single investigator to ensure consistency.

### 2.5. Echocardiographic examination

**Figure 1.**
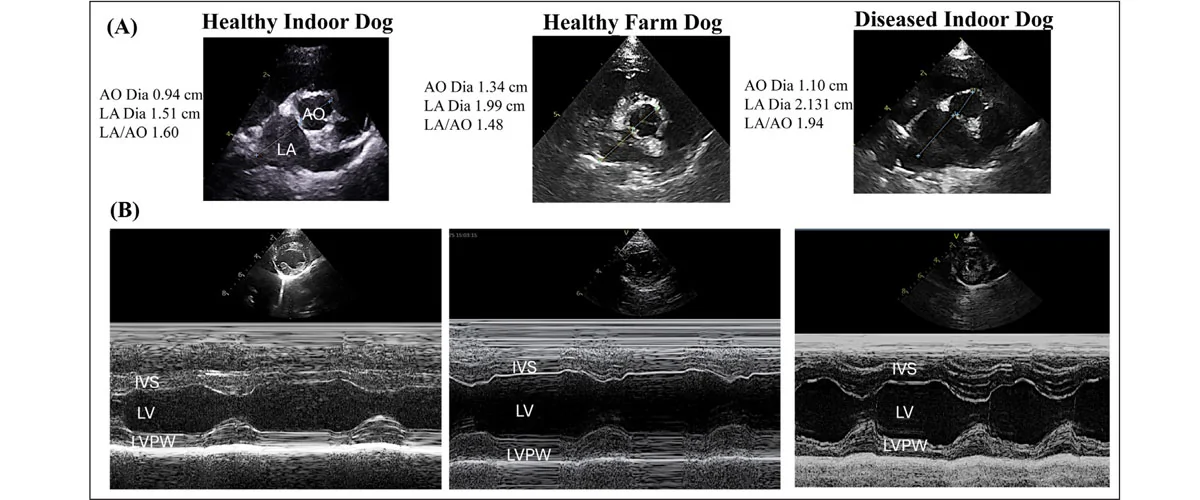
Echocardiographic images from healthy indoor (control), healthy farm, and diseased indoor dogs. (A) Right parasternal short-axis view at the level of the heart base demonstrating measurement of the left atrial (LA) diameter and aortic (AO) diameter used to calculate the LA/AO ratio in three groups: control dog, active farm dog, and indoor dog. (B) Right parasternal short-axis M-mode echocardiographic images obtained at the level of the left ventricle. Measurements include the interventricular septum (IVS), left ventricular internal diameter (LV), and left ventricular posterior wall (LVPW).

All dogs underwent transthoracic echocardiographic evaluation using a Vivid 5S ultrasound system (GE Healthcare, USA) equipped with a 6-MHz phased-array transducer. Examinations were performed by a single experienced operator to minimize inter-observer variability. Dogs were gently restrained without sedation and positioned in right and left lateral recumbency. Standard two-dimensional, M-mode, color Doppler, and spectral Doppler images were obtained following a previous study and recommended veterinary echocardiographic guidelines [9, 10, 11]. Right parasternal long-axis and short-axis views, as well as left apical views, were acquired to evaluate cardiac chamber dimensions, ventricular wall thickness, and valvular function. Measurements of left ventricular dimensions, relative wall thickness, and fractional shortening were obtained from M-mode recordings at the level of the papillary muscles. Left atrial size and aortic root diameter were measured from the right parasternal short-axis view at the level of the heart base to calculate the LA/AO ratio, as shown in Figure 1. All images and cine loops were digitally stored for offline analysis.

### 2.6. Diagnosis of mitral valve regurgitation

Dogs were examined echocardiographically for MR if an eccentric systolic jet or multiple systolic jets were documented within the LA in the right parasternal long-axis four-chamber view or left apical four-chamber view using color Doppler mapping. Single, narrow, central regurgitation jets that extended to less than approximately 10% of the LA were not classified as MR. The presence or absence of MR was recorded; the echocardiographic images were reviewed at a separate time point and without knowledge of the dog’s identity by a single observer (SP), blinded to the dog’s identity and auscultation results at the time of echocardiographic analysis.

### 2.7. Statistical analysis

Descriptive statistics were used to summarize data from the overall study population. Continuous variables were assessed for normality using the Shapiro–Wilk test. Normally distributed data are presented as mean ± standard deviation (SD), whereas non-normally distributed data are presented as median and interquartile range (IQR). Age was recorded in years. Diagnostic performance measures, including sensitivity, specificity, positive predictive value, and negative predictive value, were calculated to determine the ability of a left apical systolic murmur to detect mitral regurgitation (MR). The influence of murmur grade on these diagnostic indices was also evaluated. Comparisons between groups were performed using the independent samples t-test for normally distributed variables and the Mann–Whitney U test for non-normally distributed variables. Associations among activity level, age, and prevalence of MR were assessed using Spearman’s rank correlation coefficient. Statistical significance was defined as *p* < 0.05.

## 3. Results

A total of 39 dogs were included in the study, comprising 10 healthy indoor dogs, 14 active farm dogs, and 15 indoor dogs with structural heart disease. General characteristics, electrocardiographic, and echocardiographic findings are summarized in Tables 1, 2, and 3, respectively. Significant differences in age and body weight were observed among groups, with farm dogs being younger and heavier than indoor groups (*p* < 0.05). The median age of all dogs was 7 years; the median weight was 12.55 kg. Sixteen dogs (41%) were males. Left-sided systolic heart murmurs were detected in 22/39 (56.4%) of dogs. Mitral valve regurgitation was present in 14/39 (35.9%) dogs, and no murmur was heard in 24 dogs (65.5%). Median intensity of left-sided systolic heart murmurs was 3/6. Dogs with MR were older (9 years) than dogs without MR (4.5 years), *p* < 0.05. In this study, female dogs were more likely to have MR, with 8/14 (57.1%) vs. 6/14 (42.9%) males. Heart sound findings, heart rate, and electrocardiographic measurements recorded at rest are summarized in Table 2. Cardiac murmurs were detected in 17 of 39 dogs (43.59%). No murmurs were identified in the control group. In contrast, murmurs were present in 6 of 14 farm dogs (42.85%) and in 11 of 15 indoor dogs (73.33%). Resting heart rate differed significantly among the study groups (*p* < 0.05). Farm dogs had the lowest mean heart rate (100.8 ± 21.6 bpm), whereas indoor dogs demonstrated the highest value (120.7 ± 16.8 bpm). The control group showed an intermediate heart rate (117.5 ± 21.6 bpm). Electrocardiographic intervals were comparable across groups. The PR interval averaged 0.13 ± 0.02 sec for the total population and did not differ significantly between groups (*p* = 0.5182). Similarly, QRS duration remained consistent at approximately 0.04 sec in all groups (*p* = 0.1782). The QT interval also showed minimal variation, with mean values close to 0.20 sec (*p* = 0.5532). Mean electrical axis (MEA) values ranged from 58.33 ± 16.75 degrees in indoor dogs to 67.86 ± 23.05 degrees in farm dogs. However, these differences were not statistically significant (*p* = 0.6640). Overall, electrocardiographic parameters were within expected physiological ranges in all groups.

**Table 1.** Clinical characteristics of the study animals.

Parameters	Total (N = 39)	Healthy indoor dogs (N = 10)	Healthy farm dogs (N = 14)	Diseased indoor dogs (N = 15)	*p*-value
Age (years)	6.82 ± 4.02	9.0 ± 2.75	3.5 ± 2.38	9.0 ± 3.68	< 0.001
Body weight (kg)	14.08 ± 7.88	8.99 ± 5.80	18.33 ± 8.28	13.09 ± 6.20	0.033
Male (number (%))	16/39 (41.02%)	3/10 (30.0%)	8/14 (57.14%)	5/15 (33.33%)	–

Data are presented as mean ± SD for continuous variables and as number (%) for categorical variables. *p*-values for continuous variables were calculated using one-way ANOVA across groups. Post hoc analysis (Tukey’s test) identified a significant difference in age and body weight between indoor dogs and farm dogs (*p* < 0.05).

**Table 2.** Heart sound, heart rate, and electrocardiographic parameters of the study animals at rest.

Parameters	Total (N = 39)	Healthy indoor dogs (N = 10)	Healthy Farm dogs (N = 14)	Diseased indoor dogs (N = 15)	*p*-value
Heart murmur (number (%))	17/39 (43.59%)	0/10 (0.0%)	6/14 (42.85%)	11/15 (73.33%)	–
Heart rate (bpm)	112.5 ± 21.9	117.5 ± 21.6	100.8 ± 21.6^a^	120.7 ± 16.8^a^	< 0.05
PR (sec)	0.13 ± 0.02	0.13 ± 0.01	0.13 ± 0.13	0.13 ± 0.01	0.5182
QRS (sec)	0.04 ± 0.01	0.04 ± 0.01	0.04 ± 0.0	0.04 ± 0.01	0.1782
QT (sec)	0.20 ± 0.01	0.20 ± 0.01	0.19 ± 0.01	0.20 ± 0.0	0.5532
MEA (degree)	64.64 ± 20.78	63.75 ± 17.98	67.86 ± 23.05	58.33 ± 16.75	0.6640

Data are presented as mean ± SD. ^a^, indicates significant differences between groups based on one-way ANOVA with Tukey post hoc testing. Values sharing at least one superscript are not significantly different. Statistical significance set at *p* < 0.05.

**Table 3.** Echocardiographic parameters of the study animals at rest.

Parameters	Healthy indoor dogs (N = 10)	Healthy farm dogs (N = 14)	Diseased indoor dogs (N = 15)	*p*-value
LA/AO ratio	1.55 ± 0.22^a^	1.45 ± 0.14^b^	1.74 ± 0.18^ab^	< 0.001
LA Volume (ml)	5.89 ± 3.89^a^	9.85 ± 4.06	10.84 ± 4.57^a^	< 0.05
MPA/AO	0.93 ± 0.09	0.96 ± 0.07	1.0 ± 0.16	0.4638
LV Mass (gm)	47.22 ± 36.62^a^	135.11 ± 68.2^a^	61.07 ± 43.2^a^	< 0.0001
LVIDdN (cm)	1.48 ± 0.11^a^	1.80 ± 0.41^ab^	2.12 ± 0.49^b^	< 0.0001
RWT (cm)	0.53 ± 0.08^a^	0.41 ± 0.05^a^	0.47 ± 0.12	< 0.001
FS (%)	35.84 ± 9.28^a^	27.13 ± 3.50^a^	31.04 ± 6.19	< 0.05
MV E/A ratio	1.13 ± 0.43	0.93 ± 0.35	1.06 ± 0.13	0.3260
TV E/A ratio	0.89 ± 0.14	0.90 ± 0.09	0.82 ± 0.11	0.1437

Data are presented as mean ± standard deviation (SD). Superscripts (^a, b^) indicate significant differences between groups based on one-way ANOVA with Tukey post hoc testing. Values sharing at least one superscript are not significantly different. Statistical significance set at *p* < 0.05.

Echocardiographic parameters are summarized in Table 3. Among the study groups, several parameters describing cardiac structure were significantly different. The left atrial to aortic ratio (LA/Ao) differed significantly among groups (*p* < 0.001). Indoor dogs with structural heart disease had the highest LA/Ao values, which were significantly greater than those of farm dogs (*p* < 0.05), while healthy indoor dogs showed intermediate values. Similarly, left atrial volume differed between groups (*p* = 0.021), with indoor dogs with structural heart disease having significantly larger volumes compared with healthy indoor dogs (*p* < 0.05). Farm dogs showed intermediate values that were not significantly different from either group. Atrial volume in the control group was the smallest (5.89 ± 3.89 ml) compared to the larger values in both farm and indoor dogs. The mean value of the main pulmonary artery to aortic ratio (MPA/AO) was not significantly different between groups (*p* = 0.4638), with values remaining close to unity across all populations. Left ventricular mass was significantly different at group levels (*p* < 0.0001). The mean value was 47.22 ± 36.62 in the control group, while post hoc analysis revealed that the measurements of ventricular mass from farm and indoor groups were significantly different. Normalized left ventricular internal diameter in diastole (LVIDdN) differed significantly across groups (*p* < 0.001). Indoor dogs with structural heart disease had the highest values, followed by farm dogs, and then healthy indoor dogs; all pairwise comparisons were statistically significant (*p* < 0.05). Relative wall thickness (RWT) also differed significantly among groups (*p* < 0.001). Farm dogs had significantly lower RWT compared with healthy indoor dogs (*p* < 0.05), while indoor dogs with structural heart disease showed intermediate values that were not significantly different from the other groups. Left ventricular mass differed significantly between groups (*p* < 0.001). Farm dogs had higher absolute LV mass compared with both healthy indoor dogs and indoor dogs with structural heart disease (*p* < 0.05). However, LV mass was not indexed to body size. Therefore, interpretation should be made cautiously. The Fractional Shortening (FS) had a statistically significant difference (*p* < 0.05). Healthy farm dogs had the lowest value of mean FS, while that was the highest among the control group. Indoor dogs had intermediate systolic function. The parameters of diastolic filling were the same between groups. The mitral valve E/A ratio (*p* = 0.3260) and the tricuspid valve E/A ratio (*p* = 0.1437) were not significantly different.

**Figure 2.**
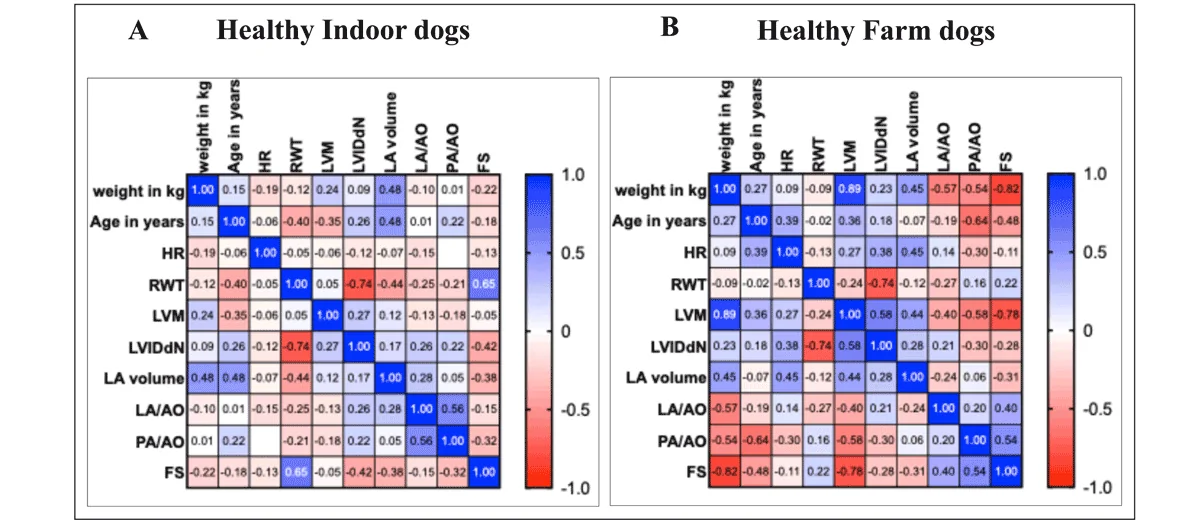
Correlation analysis of clinical and echocardiographic variables in indoor and farm dogs. (A) Heatmap, Spearman correlation coefficients of body weight, age, heart rate (HR), and echocardiographic variables in healthy indoor dogs. (B) Heatmap showing correlations among the same variables in healthy farm dogs with active lifestyles. The blue color means a positive correlation. Red color means negative correlation. Data included in Spearman correlation coefficients (ranging from -1.0 to 1.0). The variables investigated in the investigation included body weight, age, heart rate (HR), relative wall thickness (RWT), left ventricular mass (LVM), normalized left ventricular internal diameter in diastole (LVIDdN), left atrial (LA) volume, left atrial to aortic root ratio (LA/AO), pulmonary artery to aortic ratio (PA/AO), and fractional shortening (FS).

Correlation analysis (Figure 2) revealed different patterns between healthy farm dogs and indoor dogs. In active farm dogs, body weight showed a strong positive correlation with left ventricular mass (LVM; *r* = 0.89) and a moderate positive relationship with left atrial (LA) volume (*r* = 0.45). Fractional shortening (FS) demonstrated a strong negative correlation with body weight (*r* = –0.82) and LVM (*r* = –0.78). Relative wall thickness (RWT) showed a strong negative association with normalized left ventricular internal diameter (LVIDdN; *r* = –0.74). Age was moderately negatively correlated with PA/AO (*r* = –0.64) and weakly negatively correlated with FS (*r* = –0.48). In addition, PA/AO displayed a moderate positive correlation with FS (*r* = 0.54). LA/AO showed moderate negative correlations with body weight (*r* = –0.57) and LVM (*r* = –0.40). In healthy indoor dogs, fewer strong relationships were observed. RWT showed a strong negative correlation with LVIDdN (*r* = –0.74) and a strong positive correlation with FS (*r* = 0.65). Age demonstrated moderate positive correlations with LA volume (*r* = 0.48) and LVIDdN (*r* = 0.26), while a moderate negative association was observed between age and RWT (*r* = –0.40). LA/AO showed a moderate positive correlation with PA/AO (*r* = 0.56). FS showed moderate negative correlations with LVIDdN (*r* = –0.42) and LA volume (*r* = –0.38). Overall, healthy farm dogs exhibited stronger correlations between body size and cardiac structural variables, whereas healthy indoor dogs showed more limited associations, primarily involving ventricular geometry and systolic function.

## 4. Discussion

This study evaluated cardiovascular characteristics of dogs from various lifestyle categories and populations using clinical examination, electrocardiography, and echocardiography. Cardiac structure and heart rate differences between groups were identified. Heart rates of healthy farm dogs were lower than those of healthy indoor dogs. The parasympathetic tone is most often associated with increased sympathetic output in conditioned animals. Heart rate is also often predicted by body size in dogs. In this work, farm dogs were significantly heavier, and a slower heart rate may largely reflect body size-based scaling rather than exercise training [12]. Earlier research in trained dogs found reduced resting heart rate and increased stroke volume following training, though these improvements cannot be verified without a controlled evaluation of exercise.

As for cardiac structure, healthy farm dogs showed higher normalized LVIDdN and lower RWT with respect to the cardiac structure of the left ventricular chambers. This arrangement conforms to eccentric remodeling associated with sustained volume-loading patterns and has been reported in endurance-trained animals and in the athlete’s heart hypothesis [13, 14, 15]. Cardiac structure and function in the setting of exercise have been shown to alter cardiovascular structure and function, including chamber size, circulatory efficiency, and body fat loss. The healthy farm dog populations were significantly different in age and body weight, and presumably healthy farm breeds carried naturally larger cardiac widths. Thus, the differences in ventricular geometry studied are probably shaped by a combination of body size and stage of development, breed and activity level, rather than by exercise alone.

In contrast, dogs with structural heart disease (diseased indoor dogs) demonstrated increased left atrial size and abnormal functional indices, consistent with pathological remodeling. These results are consistent with earlier findings describing cardiac change attributed to myxomatous mitral valve disease, a condition closely associated with aging, increased prevalence of murmurs, and structural changes in diseased indoor dogs, which may be influenced by age-related conditions such as MMVD [16, 17, 18]. So, the increased rate of murmurs and atrial enlargement within this group was attributed more to age-related cardiac disease than reduced activity.

A key finding involved the overlap of murmur detection between groups. Such cardiac murmurs were detected in dogs with structural disease and in certain clinically healthy animals. Functional systolic murmurs can even arise in dogs with structurally normal hearts, and many are attributed to altered blood flow velocities, hematologic factors, and cardiac output [19, 20, 21]. Such murmurs have also been described in young or active dogs, and it was noted that these murmurs may be of no underlying pathology. This observation emphasizes the challenge of auscultation all by itself and further supports the potential of echocardiography to differentiate between physiological and pathological states.

Electrocardiographic data were generally uniform across groups, with no significant differences in conduction intervals or the electrical axis. Echocardiography also showed structural changes, but there were no detectable changes in conduction. Previous research has also shown that early structural remodeling may occur without major electrocardiographic abnormalities [22].

The sample size was relatively small, and the study population comprised client-owned dogs with widely variable conditions. The differences in breed, body size, and age were significant confounders that affected cardiac structure and function. Despite normalizing indices, the influence of body size remains unaccountable. Moreover, activity level was classified based on environmental context rather than objectively measured. Actual exercise intensity and duration alone were neither quantified nor ascertained, preventing a direct link between exercise and cardiac adaptation. In this cross-sectional design, the interpretation of effects is further limited as temporal alterations in cardiac anatomy cannot be assessed. Future research should include a more evenly distributed, larger population with a match in age, body mass, and breed. Objective measurement of physical activity, such as via activity-monitoring devices, would certainly facilitate the evaluation of exercise exposure. Longitudinal designs would also help assess whether structural changes are consistent with stable physiological adaptation and/or progression across time. Other imaging modalities may offer information on myocardial structure and function, such as three-dimensional echocardiography or cardiac magnetic resonance imaging.

Results for cardiovascular parameters showed significant differences in heart rate and cardiac structure between dogs across lifestyle categories. In the present study, eccentric ventricular remodeling and lower resting heart rates were detected in active farm dogs, and findings consistent with pathological remodeling were reported in dogs with structural heart disease. However, these findings should be understood as associations rather than direct consequences of exercise, as they are affected by age, body size, and population heterogeneity. The thorough evaluation of cardiovascular status should focus on echocardiography in this population to avoid misinterpreting physiologic variation as disease in dogs with murmurs or presumed cardiac enlargement.

## 5. Conclusions

In conclusion, while healthy farm dogs exhibited cardiac characteristics consistent with eccentric remodeling, these findings should be interpreted as associations rather than evidence of exercise-induced adaptation. Multiple confounding factors likely contributed to the observed differences. Careful integration of clinical examination and echocardiography is essential when evaluating cardiac findings in dogs with diverse lifestyles.

## Data Availability

The data that support the findings of this study are available from the corresponding author upon reasonable request.

## List of abbreviations

ECG, Electrocardiography; LV, left ventricle; LVID, left ventricular internal dimension; LVIDdN, normalized left ventricular internal diameter in diastole; IVS, interventricular septum; LVPW, left ventricular posterior wall; LA, left atrium; AO, Aorta; HR, heart rate; RWT, relative wall thickness; LVM, left ventricular mass; PA/AO, pulmonary artery-to-aortic ratio; FS, fractional shortening; bpm, beats per minute; cm, centimeter; kg, kilogram; ml, milliliter; MHz, megahertz; mm/sec, millimeters per second; mV, millivolt; sec, seconds.
